# Should Nurses Smoke?

**Published:** 1921-04-02

**Authors:** 


### The Editor's Letter-Box.

### Should Nurses Smoke?

*To the Editor of* The Hospital.

Sir,—Miss Gladys M. E. Leigh is usually logical. Not so, it appears to me, on this occasion.

Surely an important function of a domestic journal is to discuss domestic affairs. A quite fitting and useful topic for a teachers' journal would be—"Should Women Teachers Smoke?" Moreover, whether or not nurses are "a peculiar people," it is impossible to dissociate the individual from his or her occupation. Suppose I inquire "Should nurses frequent public-houses?" will the writer reply that they are "entitled to exercise the privileges of ordinary citizenship"?

I was at pains to shut this question outside the region of ethics, into which, by implication, your correspondent throws it back. May I point out that, inside or out, many things which for nurses as ordinary citizens are lawful are not expedient? Smoking is lawful, and it is either expedient or otherwise. It is only by free discussion that a wholesome public opinion can be fostered.—Yours faithfully,

Ierne.

